# *Gad1* knock-out rats exhibit abundant spike-wave discharges in EEG, exacerbated with valproate treatment

**DOI:** 10.3389/fneur.2023.1243301

**Published:** 2023-09-26

**Authors:** Dongyu Liu, Kazuyuki Fujihara, Yuchio Yanagawa, Hajime Mushiake, Tomokazu Ohshiro

**Affiliations:** ^1^Department of Physiology, Graduate School of Medicine, Tohoku University, Sendai, Japan; ^2^Department of Psychiatry and Neuroscience, Graduate School of Medicine, Gunma University, Maebashi, Japan; ^3^Department of Genetic and Behavioral Neuroscience, Graduate School of Medicine, Gunma University, Maebashi, Japan

**Keywords:** animal model of absence epilepsy, gamma-aminobutyric acid (GABA), glutamate decarboxylase (GAD), reticular thalamic nucleus, valproate

## Abstract

**Objective:**

To elucidate the functional role of gamma-aminobutyric acid (GABA)-ergic inhibition in suppressing epileptic brain activities such as spike-wave discharge (SWD), we recorded electroencephalogram (EEG) in knockout rats for *Glutamate decarboxylase 1* (*Gad1*), which encodes one of the two GABA-synthesizing enzymes in mammals. We also examined how anti-epileptic drug valproate (VPA) acts on the SWDs present in *Gad1* rats and affects GABA synthesis in the reticular thalamic nucleus (RTN), which is known to play an essential role in suppressing SWD.

**Methods:**

Chronic EEG recordings were performed in freely moving control rats and homozygous knockout *Gad1* (–/–) rats. Buzzer tones (82 dB) were delivered to the rats during EEG monitoring to test whether acoustic stimulation could interrupt ongoing SWDs. VPA was administered orally to the rats, and the change in the number of SWDs was examined. The distribution of GABA in the RTN was examined immunohistochemically.

**Results:**

SWDs were abundant in EEG from *Gad1* (–/–) rats as young as 2 months old. Although SWDs were universally detected in older rats irrespective of their *Gad1* genotype, SWD symptom was most severe in *Gad1* (–/–) rats. Acoustic stimulation readily interrupted ongoing SWDs irrespective of the *Gad1* genotype, whereas SWDs were more resistant to interruption in *Gad1* (–/–) rats. VPA treatment alleviated SWD symptoms in control rats, however, counterintuitively exacerbated the symptoms in *Gad1* (–/–) rats. The immunohistochemistry results indicated that GABA immunoreactivity was significantly reduced in the somata of RTN neurons in *Gad1* (–/–) rats but not in their axons targeting the thalamus. VPA treatment greatly increased GABA immunoreactivity in the RTN neurons of *Gad1* (–/–) rats, which is likely due to the intact GAD2, another GAD isozyme, in these neurons.

**Discussion:**

Our results revealed two opposing roles of GABA in SWD generation: suppression and enhancement of SWD. To account for these contradictory roles, we propose a model in which GABA produced by GAD1 in the RTN neuronal somata is released extrasynaptically and mediates intra-RTN inhibition.

## 1. Introduction

An absence seizure is a non-convulsive epilepsy accompanied by a spike-wave discharge (SWD) that appears suddenly in a patient's electroencephalogram (EEG) and lasts for several seconds (1–3). Human patients temporarily lose consciousness while paroxysmal activity emerges in the EEG. Therefore, the appearance of SWDs in an EEG is a useful clinical observation for the diagnosis of absence epilepsy. SWDs can be identified by a characteristic composite waveform consisting of a primary oscillation lasting up to 30 s (frequency: approximately 3 Hz) and large spikes superimposed on and in phase with the oscillation (1, 2). Genetic rat models for SWD have long been established (4–6); these model rats exhibit SWDs reproducibly in EEGs, with dominant oscillation frequencies of approximately 7–12 Hz (5, 7). Common laboratory rats such as Brown Norway, Long-Evans, and even wild-caught rats were recently demonstrated to exhibit abundant SWDs in EEGs (8, 9). Although SWDs in these rats are usually accompanied by the absence of seizure-like symptoms such as behavioral immobility, both naïve and genetic model rats can behaviorally detect and respond appropriately to sensory or reward cues presented when an SWD appeared on their EEG (9–12). Therefore, whether SWDs in rodents represent pathological or physiological brain activity remains controversial (7–9, 13, 14).

SWDs originate primarily within the reticular thalamic nucleus (RTN)–thalamus–cortex network (15–19). The RTN consists of GABAergic neurons that surround and innervate the thalamus, providing synaptic inhibition to thalamocortical (TC) relay neurons (20). The firing of RTN neurons elicits hyperpolarization in TC neurons, which in turn activates low-threshold T-type Ca^2+^ channels and evokes post-inhibitory rebound bursting in TC neurons (21). These TC neurons project axon collaterals back to the RTN; the re-excitation of RTN neurons leads to recurrent intra-thalamic oscillation, i.e., SWD (19).

Gamma-aminobutyric acid (GABA), the main inhibitory neurotransmitter, is synthesized from glutamate by two isoenzymes, namely, glutamate decarboxylase (GAD) 1 (67 kDa) and GAD2 (65 kDa) in mammalians. GAD2 is localized primarily in the inhibitory synapse and therefore plays an important role in synaptic inhibition (22, 23). *Gad2* knockout mice or rats are viable but suffer from spontaneous convulsive seizures (24–26). By contrast, GAD1 is primarily localized in the cytoplasm of GABAergic neurons (27, 28). Knockout rats for *Gad1* that were recently created through clustered regularly interspaced short palindromic repeats (CRISPR)/Cas9-mediated gene editing showed schizophrenia-like symptoms (29). However, whether these *Gad1* (–/–) homozygous knockout rats show any other neurological symptoms, such as epilepsy, remains to be determined.

In this study, we conducted EEG monitoring of *Gad1* (–/–) rats to evaluate their SWD symptoms in comparison with control rats. We attempted to interrupt ongoing SWDs through acoustic stimulation and conducted a pharmacological experiment using the anti-epilepsy drug valproate (VPA). Finally, we immunohistochemically analyzed the GABA expression level in RTN neurons to examine the effects of VPA treatment.

## 2. Materials

### 2.1. Animals

*Gad1* knockout rats were generated using CRISPR/Cas9-mediated gene editing (29) in the Long-Evans background. Knockout rats were crossed with Long-Evans rats (Japan SLC, Inc., Japan) and bred in the *Gad1* heterozygous (+/–) state in our laboratory. *Gad1* (–/–) and *Gad1* wild-type (+/+) rats were obtained from sibling mating between heterozygotes. All rats were reared under standard laboratory conditions under a 12-h/12-h light/dark cycle. All experimental procedures were approved by the Animal Care and Experimentation Committee of Gunma University and the Tohoku University Committee for Animal Research.

### 2.2. Surgery and EEG recordings

Implant surgery and EEG recording were performed as described previously (25). Briefly, rats were anesthetized with medetomidine, midazolam, and butorphanol mixture and placed on the stereotaxic apparatus. Heart rates and SpO_2_ were continuously monitored. The scalp was excised, and the parietal skull bone was exposed. Small burr holes (~0.9 mm diameter) were made bilaterally over the skull, and twisted-pair wire electrodes (114 μm diameter, TFA-coated silver wire, Med Wire, USA) and/or silver-pleated screw electrodes (M1 × 3 mm, Unique Medical, Japan) were aseptically implanted. The tips of the twisted-pair wire electrodes were inserted into the cortex at a depth of 1 mm and firmly supported through a ceramic guide tube (KyoCera, Japan). After the implant surgery, the rats were returned to the home cage and were allowed to recover from the surgery for 1 to 2 days before the first recording was made. On the EEG recording, the rat with the implant was transferred from the home cage to a clear plexiglass cage (60 × 60 × 60 cm), on whose walls two piezoelectric speakers (Digi-Key Electronics, Thief River Falls, MN, USA) were pasted. A wired head-stage amplifier (HST/8o50-G1-GR; Plexon, Inc., Dallas, TX, USA) or wireless head-stage amplifier (TBSI Wireless Neural Recording System, Global Bio Inc., Mount Laurel, NJ, USA) were used for EEG recording. Raw electrical signals were further amplified and digitized using the Omniplex multichannel recording system (Plexon). In total, 45 rats of both sexes, including 20 *Gad1* (–/–), 9 *Gad1* (+/–), and 16 *Gad1* (+/+) rats, were used in this study. The rats were separated into two age groups, younger (<2 months) and older (≥2 months), and their EEGs were examined for SWDs. Because SWDs were rare in younger *Gad1* (+/–) and *Gad1* (+/+) rats, only EEG data from older rats were further analyzed. Thus, in total, 100 h of EEG data were collected and analyzed from 10 *Gad1* (–/–) rats (age: 2–11 months), 45 h from 6 *Gad1* (+/–) rats (age: 3–12 months), and 70 h from 7 *Gad1* (+/+) rats (age: 2–12 months). EEG recording was performed for up to 5 h per day, and recording lasted for up to 3 months in a single animal. Some EEG recording sessions were videotaped to record rat behavior.

### 2.3. Acoustic stimulation experiment

In the acoustic stimulation experiment, a rat was placed in a clear plexiglass cage while wearing an EEG headset. The experimenter continuously monitored the EEG for SWDs and applied a series of acoustic stimulations from piezoelectric speakers (dominant frequency: 2 kHz; loudness: 82 dB [measured at the cage center]; ambient loudness: 44 dB) as soon as an SWD appeared on the monitor. The recording was started without acoustic stimulation for 1–2 h and continued for another 1–2 h with acoustic stimulation. In total, 22 h of EEG recording data were obtained from five *Gad1* (–/–) rats (age: 2–10 months), as well as 28 h of EEG recording data from two *Gad1* (+/–) rats and four *Gad1* (+/+) rats (age: 3–9 months). Because *Gad1* (+/–) and *Gad1* (+/+) rats yielded similar results, the data from these genotype groups were merged and used as control group data.

### 2.4. Offline SWD detection

**Figure 1:**
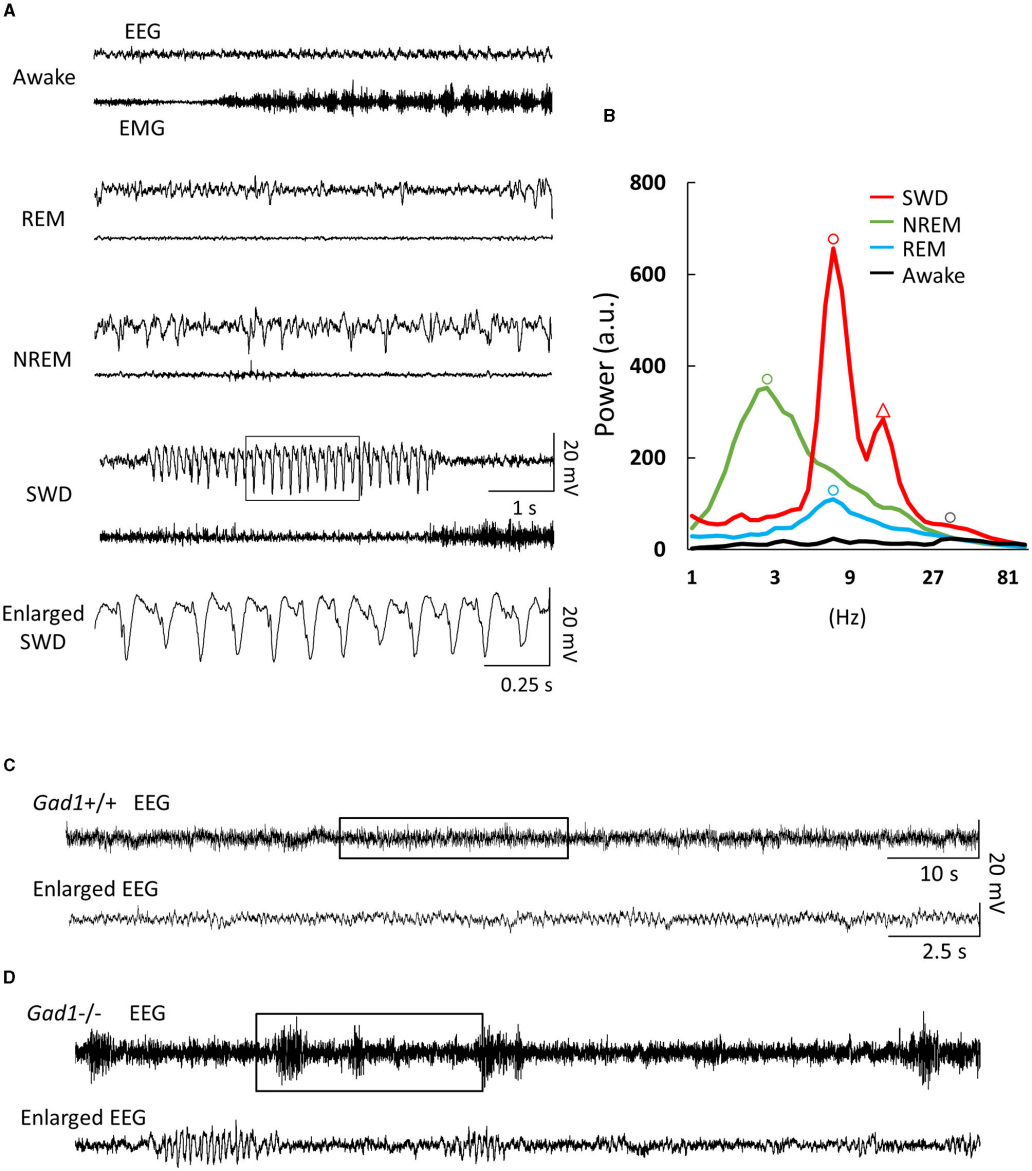
Electroencephalogram (EEG) and electromyogram (EMG) patterns. **(A)** EEG/EMG patterns from a *Gad1* homozygous (–/–) knockout rat. Similar patterns were observed in both *Gad1* heterozygous (+/–) and wild-type (+/+) rats. In the awake state, the animals showed a rapid, small-amplitude EEG pattern and a highly active EMG pattern. Rapid-eye-movement (REM) sleep was associated with complete muscle atonia, despite the active EEG pattern. Non-REM (NREM) sleep was associated with a slow, large-amplitude EEG pattern. Spike-wave discharges (SWDs) (framed section of the EEG, enlarged at the bottom) appeared suddenly while the rats were awake, although they tended to remain still. **(B)** EEG power spectrum with arbitrary units (a.u.). Peaks are indicated by circles. The dominant frequencies for SWD, NREM sleep, REM sleep, and wakefulness were 8, 3, 7, and 30 Hz, respectively. The SWD power spectrum also showed a second prominent peak (triangle) at the first harmonic, i.e., 16 Hz, reflecting a spiky waveform shape. **(C)** EEG pattern from a 7-week-old *Gad1* (+/+) rat. Few SWDs were seen at ages of <2 months. The framed section (top) is enlarged at the bottom. **(D)** EEG patterns from a 5-week-old *Gad1* (–/–) rat showed many SWDs. The framed section (top) is enlarged at the bottom.

All EEG recording data were analyzed offline for SWDs using custom-made programs written in MATLAB code. Briefly, raw voltage signals sampled at 1 kHz were subjected to high-pass filtering (cutoff: 0.9 Hz) and wavelet-transformed (30). SWDs were extracted from the total spectral power time series data by imposing frequency thresholds at 5 and 32 Hz; this interval contained most of the SWD oscillation power (Figure 1). The extracted SWDs were checked visually, and mechanical noise caused by rats tapping the cable or chewing food pellets was excluded from the data. By our definition, a spike-train waveform lasting <1 s was not regarded as an SWD.

### 2.5. VPA treatment

We initially examined the effect of intraperitoneal (i.p.) injections of VPA (150–300 mg/kg, dissolved in 1 ml of saline) using three *Gad1* (–/–), two *Gad1* (+/–), and two *Gad1* (+/+) rats. In this pilot experiment, we observed that all became drowsy and inactive after the injection, except for one *Gad1* (+/+) rat. To avoid acute poisoning and the need for repeated i.p. administration, we next tried a milder oral formulation of the drug, using another set of rats (two *Gad1* (–/–), four *Gad1* (+/–), and two *Gad1* (+/+) rats). VPA (10 mg/ml) was dissolved in water and made available to the rats *ad libitum* for 7 days. At the end of this period, we collected the blood serum samples from these animals and outsourced them to a local biochemistry lab (Nagahama Life Science Laboratory, Shiga, Japan) for the VPA blood concentration measurement. VPA concentration was measured based on the enzyme immunoassay method (Emit2000 valproate assay, Siemens Healthcare, Japan). The average blood concentration of VPA was 40.4 ± 10.4 μg/ml (24.1–73.7; n = 8 animals). The effective blood concentration of VPA for the treatment of epilepsy in humans is 50–100 μg/ml (31). Based on these observations, we decided to adopt the oral administration of the drug for the EEG recording experiment and the GABA immunohistochemistry. For the recording experiment, a pre-medication EEG recording was performed 2–3 days before starting drug administration [control group: VPA (–)]. EEG recording was resumed on the 3rd day after the initiation of drug administration and performed for a total of 2–3 days until the 7th day of the VPA treatment [experimental group: VPA (+)] (Table 1). In total, we obtained 70 h of EEG recording data from four *Gad1* (–/–) rats (age: 5–11 months) and 68 h from four *Gad1* (+/+) rats (age: 5–12 months). The water intake and body weight were monitored during the experiment (Table 2).

**Table 1:** EEG recording time before and during the VPA treatment.

**Animal ID**	**Pre-medication**	**VPA treatment**						
	**2–3 days**	**1st day**	**2nd day**	**3rd day**	**4th day**	**5th day**	**6th day**	**7th day**
1. *Gad1* (+/+)	10 h in total	-	-	4 h	-	4 h	-	2 h
2. *Gad1* (+/+)	9 h	-	-	-	4 h	-	3 h	2 h
3. *Gad1* (+/+)	5 h	-	-	-	5 h	-	-	-
4. *Gad1* (+/+)	10 h	-	-	5 h	5 h	-	-	-
1. *Gad1* (–/–)	7 h	-	-	-	4 h	-	3 h	-
2. *Gad1* (–/–)	10 h	-	-	5 h	-	-	-	5 h
3. *Gad1* (–/–)	10 h	-	-	-	5 h	-	2 h	3 h
4. *Gad1* (–/–)	8 h	-	-	4 h	-	4 h	-	-

**Table 2:** Amounts of water/VPA intake (average) and rat's weight (range) before, during, and after VPA treatment.

**Animal ID**	**Pre-medication (2–3 days)**	**VPA treatment (7 days)**	**VPA cessation (3 days)**
1. *Gad1* (+/+)	37 ml/day, 502–510 g	25 ml/day, 489–478 g	40 ml/day, 498 g
2. *Gad1* (+/+)	22 ml/day, 282–276 g	12 ml/day, 260–252 g	27 ml/day, 275 g
3. *Gad1* (+/+)	33 ml/day, 316–324 g	30 ml/day, 302–298 g	N.D.
4. *Gad1* (+/+)	25 ml/day, 272–277 g	20 ml/day, 247–242 g	30 ml/day, 263 g
1. *Gad1* (–/–)	33 ml/day, 308–312 g	23 ml/day, 300–294 g	30 ml/day, 300 g
2. *Gad1* (–/–)	45 ml/day, 476–477 g	23 ml/day, 458–465 g	50 ml/day, 474 g
3. *Gad1* (–/–)	30 ml/day, 310–300 g	20 ml/day, 288–272 g	30 ml/day, 284 g
4. *Gad1* (–/–)	31 ml/day, 281–282 g	18 ml/day, 256–255 g	N.D.

The body weight and the water intake of the rats were decreased by valproate treatment (VPA, 10 mg/ml, per os), but recovered after cessation of VPA treatment. N.D. (no data).

### 2.6. Immunohistochemistry

Four *Gad1* (+/+) VPA (–), five *Gad1* (+/+) VPA (+), four *Gad1* (–/–) VPA (–), and four *Gad1* (–/–) VPA (+) rats were euthanized by intraperitoneal injection of pentobarbital (150 mg/kg animal) and perfused with one-half Karnovsky fixative (2% paraformaldehyde and 2% glutaraldehyde in phosphate-buffered saline). The fixed brain was immersed in 30% sucrose in 0.1 M phosphate buffer for cryoprotection and embedded in Tissue-Tek OCT compound (Sakura Finetek Japan, Tokyo, Japan). The frozen brain was cut into 10-μm-thick sections using a cryostat (CM1950, Leica, Wetzlar, Germany) and mounted onto coated slide glasses (MAS-01, Matsunami Glass, Kishiwada, Japan). Coronal sections (ca. bregma −2.7 mm) were selected to examine the RTN and neighboring thalamic nuclei. Immunostaining was performed using a STAINperfect Immunostaining Kit (ImmuSmol, Bordeaux, France), following the manufacturer's instructions. The primary antibodies used in these experiments included chicken polyclonal anti-GABA (1:500 dilution; ImmuSmol), rabbit polyclonal anti-parvalbumin (PV) (1:2,000, GTX134110, Genetex, Hsinchu, Taiwan), chicken normal serum (for negative control, 1:2,000 dilution; Jackson ImmunoResearch), and the secondary antibodies were preabsorbed Alexa Fluor-488 conjugated goat anti-chicken IgY (1:500 dilution; ab150173, Abcam, Cambridge, UK) and preabsorbed Alexa Fluor-568 conjugated goat anti-rabbit IgG (1:500 dilution; ab175695, Abcam, Cambridge, UK). For fluorescent nucleus staining, we used a CellStain−4′,6-diamidino-2-phenylindol (DAPI) solution (1:500 dilution, Dojindo Kumamoto, Mashiki, Japan). Fluorescence images were acquired using an LSM 780 laser confocal microscope (Zeiss, Oberkochen, Germany) and a 63 × oil objective lens (numerical aperture: 1.4) and analyzed using the ZEN confocal image analysis software (Zeiss) and ImageJ software (National Institutes of Health, Bethesda, MD, USA), respectively (32–34) (Supplementary Figures S1, S2).

### 2.7. Statistical analyses and graphics

Median values were compared using the Wilcoxon rank-sum, Wilcoxon signed-rank, and Kruskal–Wallis tests using the MATLAB Statistics and Machine Learning Toolbox. Multiple comparisons were performed using the *multicompare* function with Fisher's least significant difference option in the same toolbox. All plots were drawn using Excel software (Microsoft Corp., Redmond, WA, USA). In the box-whisker plot, the upper (lower) whisker was drawn from the top (bottom) of the box, up (down), to the largest (smallest) data point within 1.5-fold of the interquartile range. Outliers outside the upper and lower whiskers are not shown in the plot.

## 3. Results

### 3.1. Abundant SWDs in young *Gad1* (–/–) rats

EEG patterns in *Gad1* (–/–) rats during the awake or sleep (rapid eye movement and non-rapid eye movement) states appeared to be similar to those in normal animals (32) (Figure 1A). However, on visual inspection of the EEG data, frequent large-amplitude spiky waveforms caught the attention (Figure 1A, SWD), which lasted a few seconds and oscillated at approximately 8 Hz (Figure 1B). These features are suggestive of spike-wave discharges in rodents (5, 7). These SWDs were observed while the rats were awake but quiet or at rest after active exploration. SWDs were observed in *Gad1* (–/–) rats as early as 2 months of age, whereas they were rarely detected in *Gad1* (+/–) or *Gad1* (+/+) rats at similar ages (Figures 1C, D, Table 3). Only one out of seven rats showed SWDs (29 SWDs were detected from a total of 10 h of recording). The other six control rats did not show SWD in their EEG (a total of 175 h of recording). However, even in *Gad1* (+/+) rats, SWDs eventually prevailed in the EEG at older ages. No overt convulsive seizures or other epileptic activity were observed in *Gad1* (–/–) rats (data not shown).

**Table 3:** Proportion of rats showing SWDs sorted by age.

***Gad1* genotype**	**Younger than 2 months old**	**Older than 2 months old**
(–/–)	7/8	16/16
(+/–)	0/4	6/6
(+/+)	1/3	14/16

The rats were separated into two age groups (young and old) and examined for the presence of spike-wave discharges (SWDs) in EEG. In all, 45 rats were recorded; 4 out of 20 Gad1 (–/–) rats, 1 out of 9 Gad1 (+/–) rats, and 3 out of 16 Gad1 (+/+) rats were examined in both age groups.

**Figure 2:**
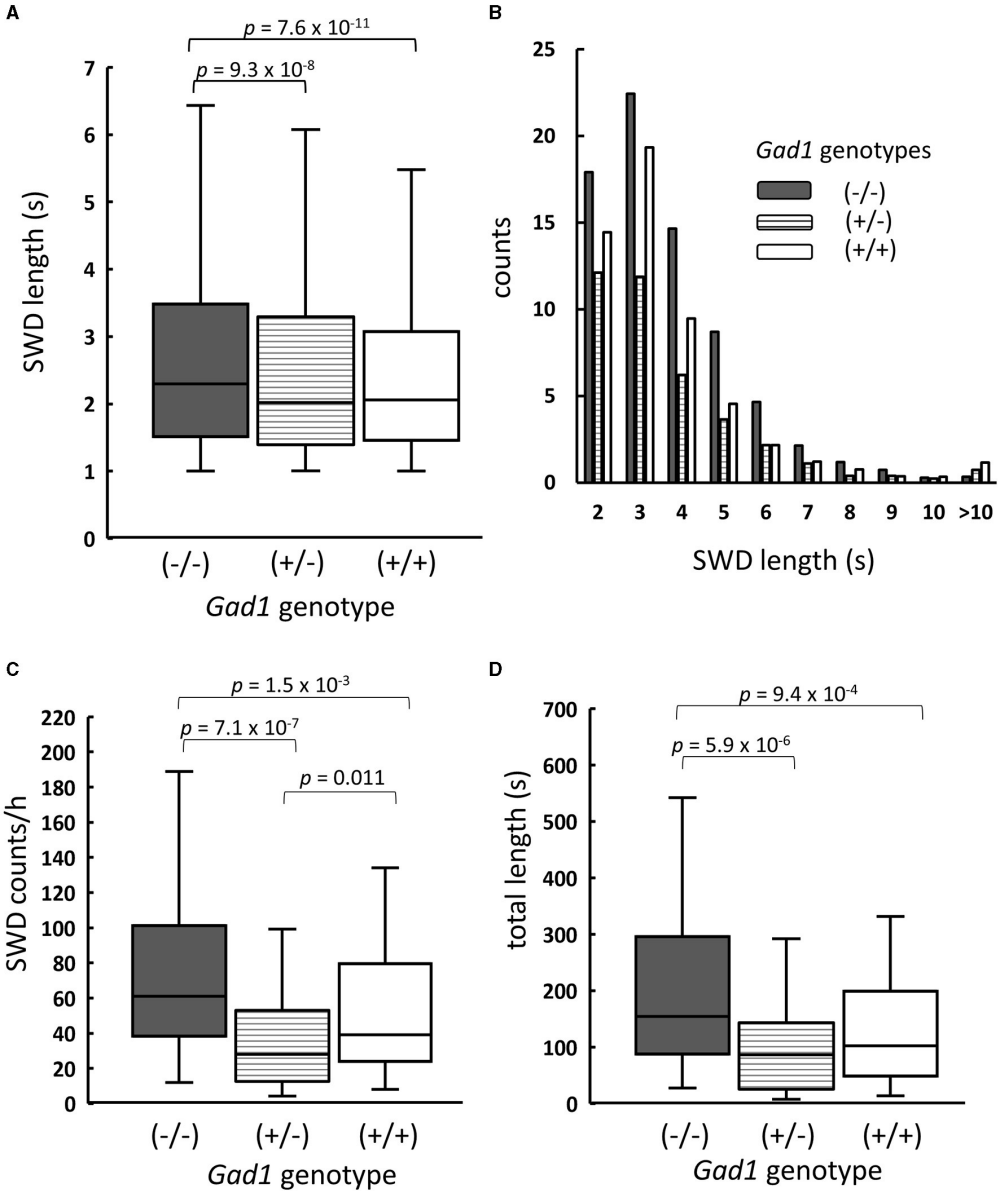
Summary of SWDs from *Gad1* (–/–) rats (shaded), *Gad1* (+/–) rats (lined), and *Gad1* (+/+) rats (unshaded). **(A)** Box and whisker plots of individual SWD lengths. The median SWD length was greatest in the *Gad1* (–/–) group. **(B)** Distribution of SWD counts per hour, sorted by length. Most SWDs lasted for 5 s. **(C)** Accumulated SWD counts per hour. The median count was highest in the *Gad1* (–/–) group. **(D)** Accumulated lengths of individual SWDs per hour. The median length was greatest in the *Gad1* (–/–) group. In the graphs, SWD data were merged within the same experimental group, and the medians of each group were compared (Wilcoxon rank-sum test), i.e., *P*-values were obtained based on events but not animals. See the text for detailed statistical information.

As SWDs were rare in young *Gad1* (+/–) and *Gad1* (+/+) rats, only SWDs from older rats were analyzed and compared among the three genotypes (Figure 2). SWDs were significantly longer in *Gad1* (–/–) rats (median: 2.3 s; *n* = 7,305 SWDs from 10 rats) than in *Gad1* (+/–) rats (2.0 s; n = 1,749 SWDs from 6 rats) or *Gad1* (+/+) rats (2.1 s; n = 3,766 SWDs from 7 rats) (Figure 2A; *P* = 4.2 × 10^−13^; Kruskal–Wallis test). The difference between *Gad1* (+/+) and *Gad1* (+/–) rats was not statistically significant (*P* = 0.41; Wilcoxon rank-sum test). The SWD length shows long-tailed distributions (Figure 2B), with the longest SWD lasting longer than 10 s. Most SWDs were shorter than 5 s in duration, regardless of genotype. The median SWD count in *Gad1* (–/–) rats (61/h) was the largest among the three genotypes (*P* = 8.3 × 10^−7^; Kruskal–Wallis test) (Figure 2C), while the median SWD in *Gad1* (+/–) rats (28/h) was significantly smaller than in *Gad1* (+/+) rats (39/h) (*P* = 0.011; Wilcoxon rank-sum test). Therefore, the total SWD time per hour was greatest in *Gad1* (–/–) rats (median: 155 s/h), which was significantly different from *Gad1* (+/–) and *Gad1* (+/+) rats (median: 86 and 102 s/h, respectively, *P* = 5.7 × 10^−6^; Kruskal–Wallis test) (Figure 2D). The total SWD time in *Gad1* (+/–) was shorter than *Gad1* (+/+) rats, however the difference was insignificant (*P* = 0.09; Wilcoxon rank-sum test). These results indicate that *Gad1* (–/–) rats had the most severe SWD phenotype and that GAD1 plays a role in suppressing SWD generation in rats.

### 3.2. Interruption of SWD by acoustic stimulations

**Figure 3:**
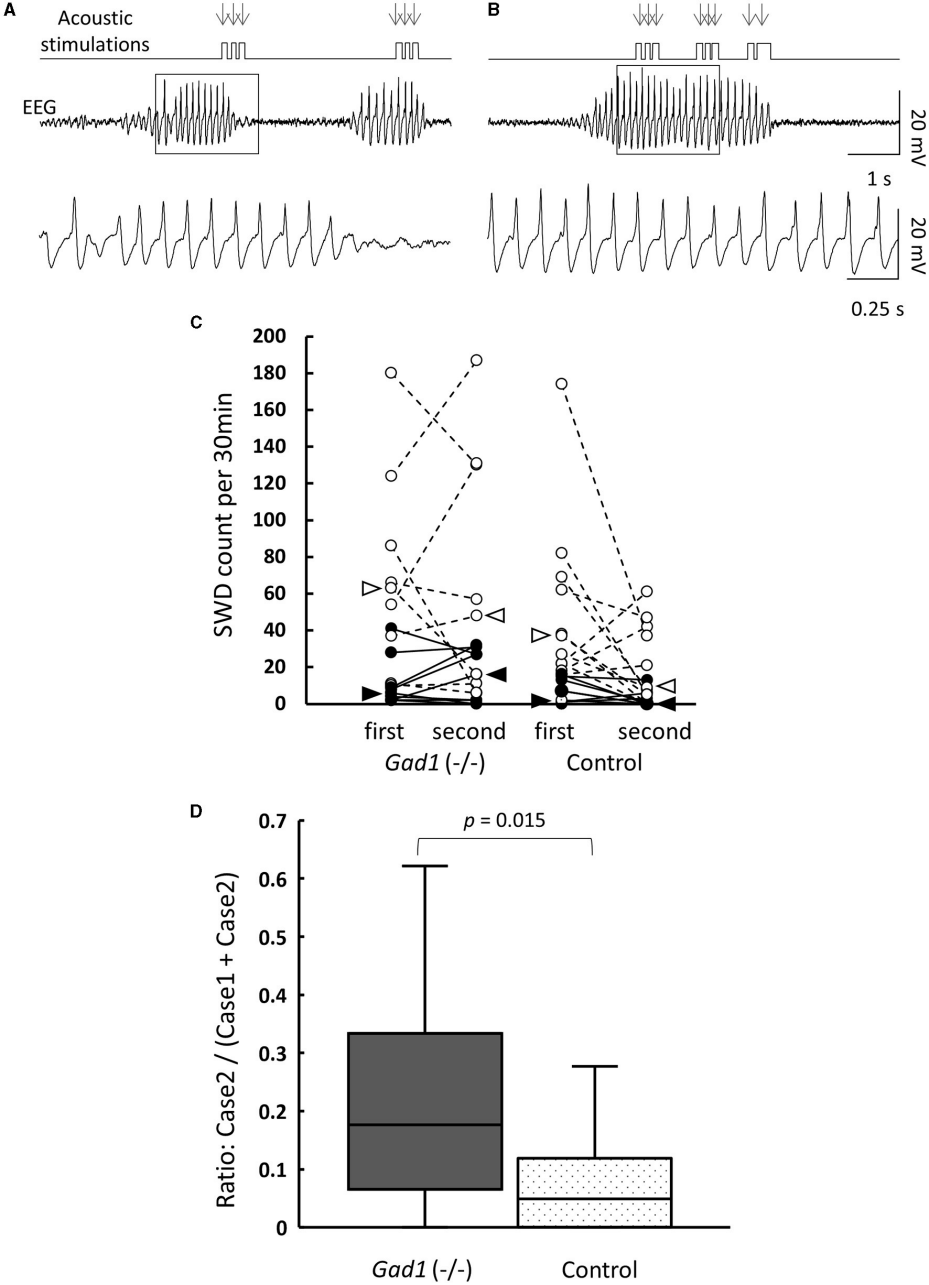
Interruption of SWDs by auditory stimuli (AS). **(A)** Case 1: SWDs on the EEG were readily interrupted by AS. The timing of AS presentation is indicated by arrows. The framed section of the SWD data is enlarged at the bottom. **(B)** Case 2: SWDs were not interrupted by AS. The framed section of the SWD data is enlarged at the bottom. Both examples are from a *Gad1* (–/–) rat. The other genotypes showed similar patterns. **(C)** SWD counts according to genotype and occurrence period (first or second half) during the 1-h recording session. Total SWD counts, including both Cases 1 and 2, are indicated by open circles, and Case 2 counts are indicated by filled circles. Circles from the same rat are connected. Medians are indicated by open arrowhead (Cases 1 and 2) or filled arrowhead (Case 2 only). **(D)** Accumulated Case 2 ratio (Case 2/[Cases 1 and 2]) sorted by genotype. *P*-values were calculated using the Wilcoxon rank-sum test.

In human patients with absence seizures, SWD appears abruptly in EEG, while the patients temporally lose consciousness and are often unaware of external sensory stimuli (1–3). Therefore, we investigated whether the rats were responsive to external acoustic stimulation when SWDs appeared in the EEG. We presented buzzer tones through speakers to awake-behaving animals during live EEG monitoring. The experimenter presented the buzzer tone as soon as an SWD was detected in the EEG displayed on a monitor. *Gad1* (–/–) and control rats responded overtly to these buzzer tones (Table 4). Most SWDs were interrupted readily by a single volley of buzzer tones (Case 1 SWD, Figure 3A), whereas the remaining SWDs were not (Case 2 SWD, Figure 3B). The appearance of SWDs in EEG was highly correlated with the inactivity of animals caused by reward withdrawal or a loss of motivation to achieve the reward during behavioral tasks (33). Because buzzer stimulations were not associated with reward and did not require a voluntary response, the SWDs observed in the rats, including in Case 2, likely reflect the animal's gradual loss of interest in, or sensory adaptation to, repeated buzzer tones (11, 33). If this is true, the number of SWDs should increase over time. To test this possibility, the SWDs were categorized into the first and second halves of the 1-h recording session. The total SWD count, including that of Cases 1 and 2, was not significantly different between the first and second halves of the session in *Gad1* (–/–) rats (median: 63 and 48 per 30 min, respectively; n = 9; *p* = 0.82, Wilcoxon signed-rank test) and control rats (median: 37 and 9 per 30 min, respectively; *n* = 11; *p* = 0.10) (Figure 3C). Similarly, the SWD counts of Case 2 were not significantly different between the first and second halves of the session in *Gad1* (–/–) rats (median: 6 and 16 per 30 min, respectively; *p* = 0.51) or control rats (median: 2 and 0 per 30 min, respectively; *p* = 0.11). We extended three recording sessions for an additional hour and observed no significant increase in SWD counts in any group (data not shown). Therefore, our results do not suggest that the SWDs were produced by sensory adaptation or a loss of motivation over time. Interestingly, the SWD count in Case 2 was significantly higher in *Gad1* (–/–) rats (median Case2/(Case1 + Case2) ratio: 18%; n = 18) than in the control group (median ratio: 5%; n = 20) (*p* = 0.015, Wilcoxon rank-sum test) (Figure 3D). These results indicate that rats responded to sensory stimuli when SWDs appeared in EEG, and *Gad1* (–/–) rats had more SWDs resistant to sensory interruption.

**Table 4:** Results of hearing ability test.

**Genotype group**	**Total**	**Responses**
*Gad1* (–/–) group	300	281
Control group	300	277

Three Gad1 (–/–) rats, one Gad1 (+/–) rats (control group), and two Gad1 (+/+) rats (control group) were presented with a short buzzer tone (300 times in each group), and their overt responses to the tone, e.g., twitching a body part (pinna, neck, whisker pad, and so forth) or interrupting their behavior (chewing pellets, exploring on the floor, and so forth) were counted. A total of 281 responses were recorded in the Gad1 (–/–) group, and 277 responses were recorded in the control group, indicating that all genotypes had similar hearing abilities.

### 3.3. Anti-epileptic drug test

**Figure 4:**
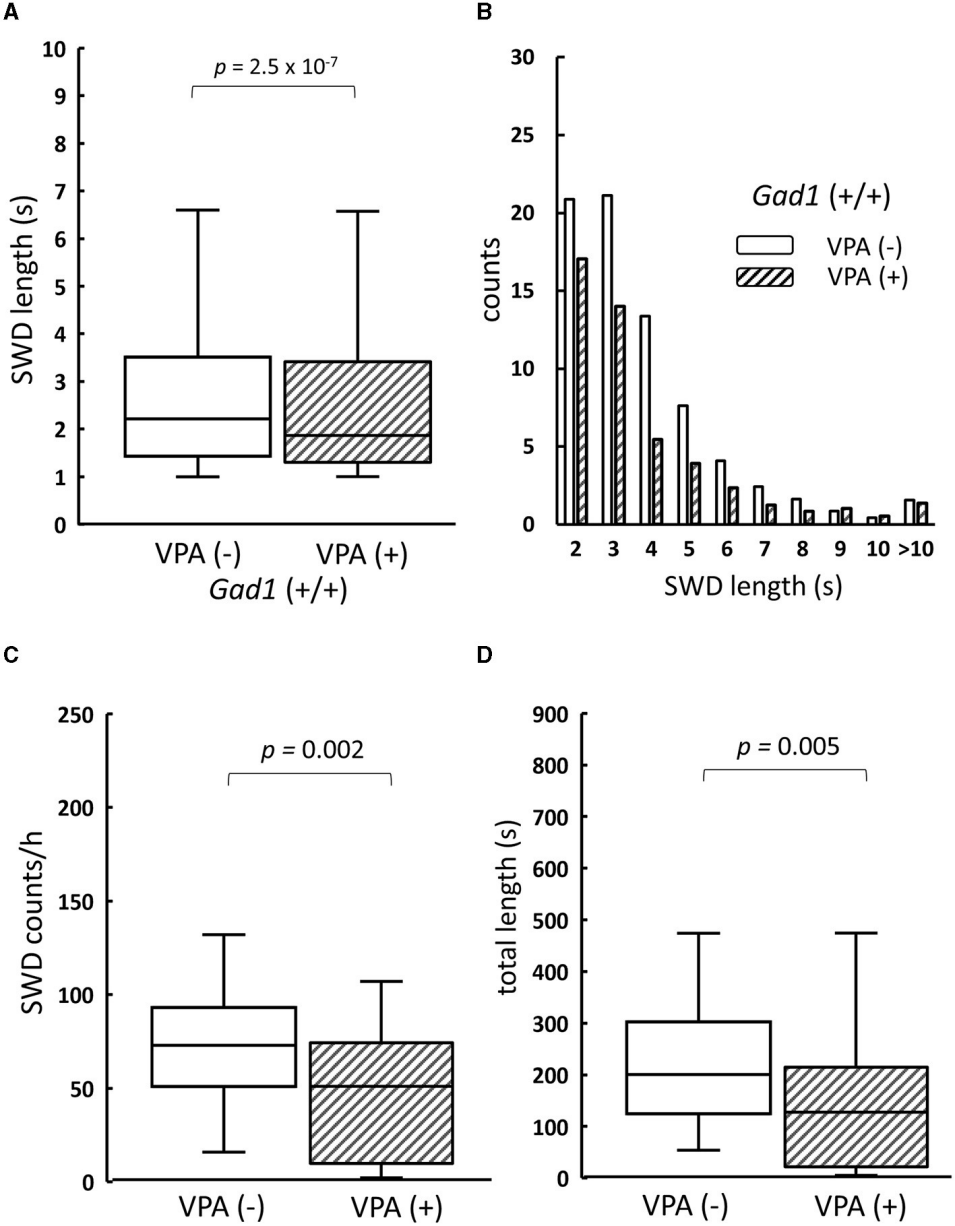
The effect of valproate treatment on SWD in *Gad1* (+/+) rats. **(A)** The median SWD length before valproate treatment [VPA(–)] was significantly decreased by VPA administration [VPA (+)]. **(B)** SWD counts per hour decreased in almost all SWD length categories following VPA administration. **(C)** The median SWD count per hour was significantly decreased by VPA treatment. **(D)** The median accumulated SWD time per hour was significantly decreased by VPA treatment. SWD data were merged within the same experimental group, and the medians of each group were compared (the Wilcoxon rank-sum test).

We next examined VPA, a medication commonly used to treat both the absence and the myoclonic seizure, which inhibits GABA degradative enzymes such as GABA transaminase (34, 35), thereby increasing the GABA level in the brain. As SWD was a common symptom in both older *Gad1* (+/+) and *Gad1* (–/–) rats, we expected that an increase in GABA caused by VPA would alleviate the SWD phenotype in both genotypes. Consistent with this expectation, the SWD phenotype in *Gad1* (+/+) rats (*n* = 4) was significantly alleviated by VPA treatment (Figure 4). The median length of individual SWDs was decreased by VPA treatment, from 2.2 s (without VPA treatment; *n* = 2,516 SWDs) to 1.9 s (with VPA treatment; n = 1,625 SWDs) (Figure 4A). A decrease in SWD length was observed over the entire range of SWD sizes (Figure 4B). The median count of SWD appearances per hour decreased from 73 to 51 (Figure 4C); thus, the accumulated SWD length per hour decreased from 200 to 127 s following VPA treatment (Figure 4D).

**Figure 5:**
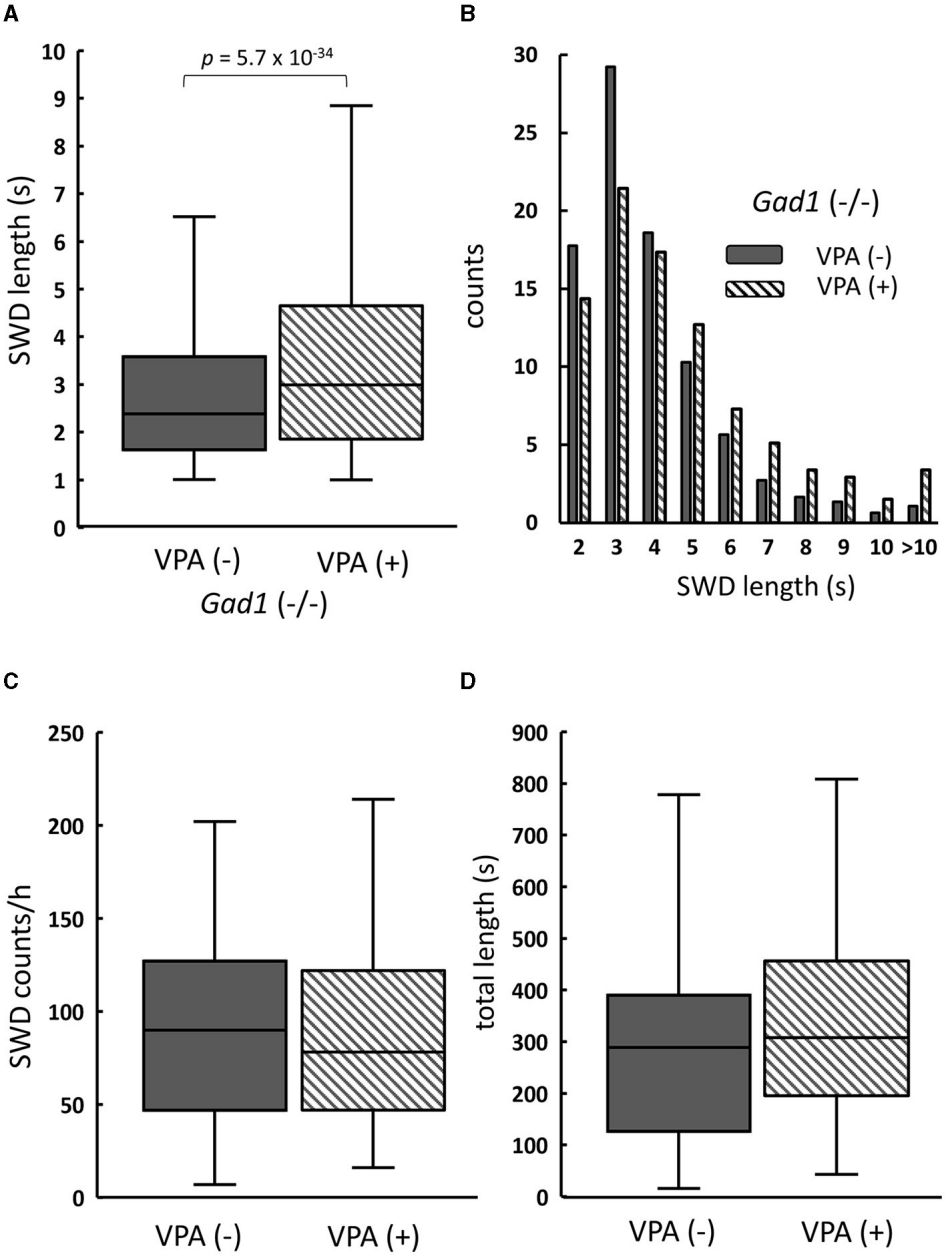
The effect of valproate treatment in *Gad1* (–/–) rats. **(A)** The median SWD length before valproate treatment [VPA(–)] was significantly increased by VPA administration [VPA(+)]. **(B)** Following VPA treatment, SWD counts per hour decreased for shorter SWDs but increased for longer SWDs. **(C)** The median SWD count per hour was slightly decreased by VPA, but the effect was not statistically significant (*P* = 0.77). **(D)** The median SWD length per hour was increased by VPA, but the effect was not statistically significant (*P* = 0.23). SWD data were merged within the same experimental group, and the medians of each group were compared (the Wilcoxon rank-sum test).

Counterintuitively, the SWD phenotype in the *Gad1* (–/–) rats (*n* = 4) was worsened by VPA treatment (Figure 5). The median SWD length without treatment (median: 2.4 s; *n* = 3,113 SWDs) increased to 3.0 s following VPA treatment (*n* = 3,132 SWDs) (Figure 5A). Notably, SWDs shorter than 4 s in duration decreased in number, whereas those lasting longer than 5 s became more frequent (Figure 5B). The number of SWD occurrences, irrespective of size, did not differ significantly between treatment groups (Figure 5C). Overall, accumulated SWD length per hour increased from 289 to 307 s; however, this increase was not significant (Figure 5D). In summary, VPA treatment exacerbated the SWD symptom in *Gad1* (–/–) rats, unlike in control rats, suggesting that a mere increase in GABA level by the drug cannot compensate for GAD1 deficiency.

### 3.4. GABA immunoreactivity in reticular thalamic nucleus

**Figure 6:**
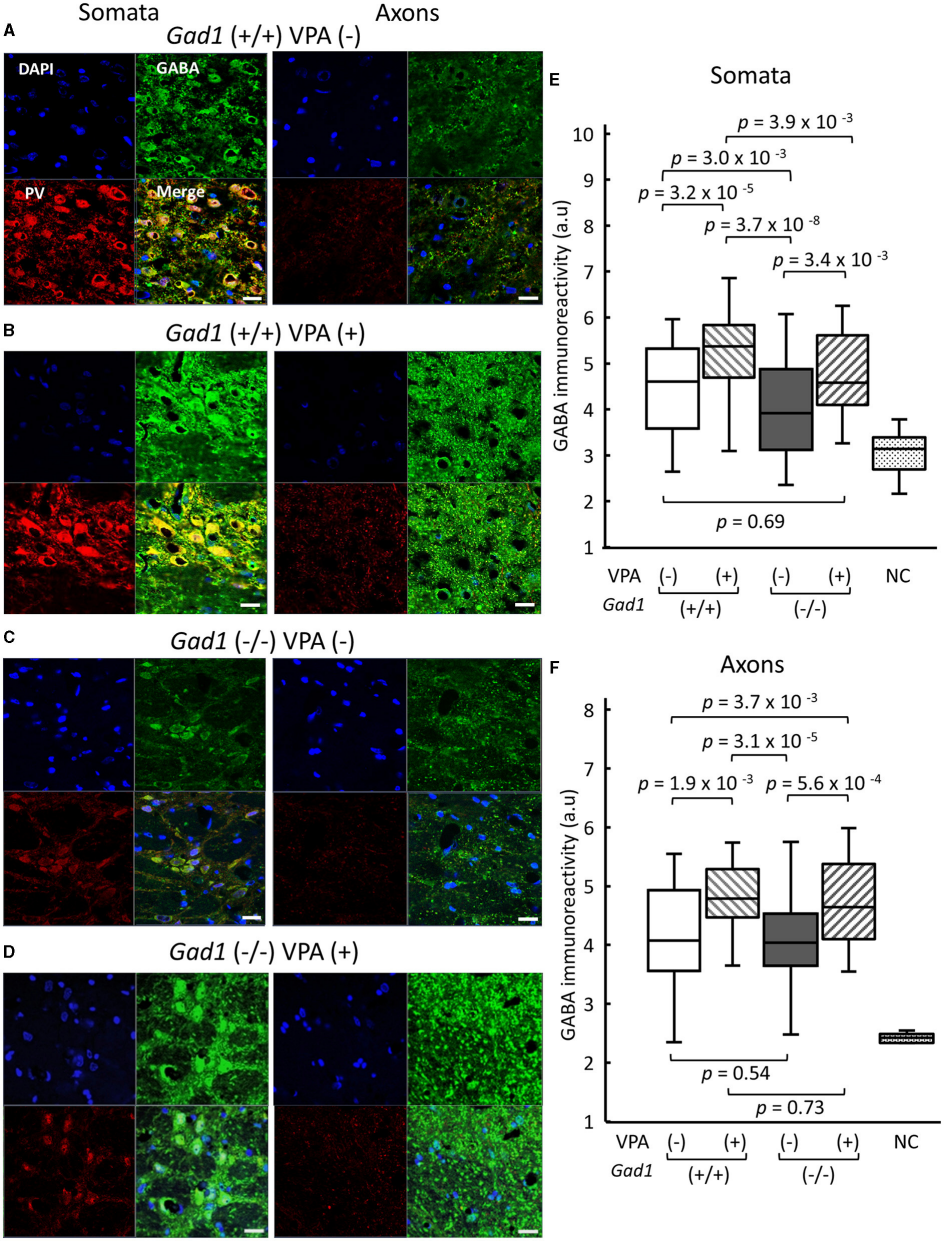
GABA immunoreactivity in neuronal soma of RTN neurons and their axons targeting the thalamus. White scale bar: 20 μm. Immunohistochemical detection of gamma aminobutyric acid (GABA, green) and parvalbumin (PV, red) in **(A)** *Gad1* (+/+) VPA (–) rats, **(B)** *Gad1* (+/+) VPA (+) rats, **(C)** *Gad1* (–/–) VPA (–) rats, and **(D)** *Gad1* (–/–) VPA (+) rat. Cell nuclei were labeled with DAPI (blue). **(E)** GABA immunoreactivity was significantly lower in the RTN neuronal soma of *Gad1* (–/–) rats without VPA treatment. **(F)** GABA immunoreactivity in the RTN axons was not significantly different between *Gad1* (–/–) and *Gad1* (+/+) rats, irrespective of VPA treatment. *P* values in the graphs were calculated by pairwise comparisons (the Wilcoxon rank-sum test). Negative control (NC) was included to indicate the background immunoreactivity level, in which chicken normal serum was used as the primary antibody in place of the chicken anti-GABA antibody.

GAD1 is abundantly expressed in GABAergic neurons in the RTN, which plays an important role in SWD generation (15–19). We finally examined GABA immunoreactivity in the RTN (Figure 6). We identified GABAergic RTN neurons and their axons through co-immunostaining with an antibody against parvalbumin (PV), a peptide marker for the GABAergic RTN neurons (36–39). In *Gad1* (+/+) rats without VPA treatment [VPA(–)], a strong GABA immunoreactivity was detected in the somata of RTN neurons (Figure 6A, left). A further enhanced GABA immunoreactivity was observed after VPA treatment [VPA(+)] (Figure 6B, left). As expected, GAD1 deficiency reduced GABA immunoreactivity in the somata (Figure 6C, left). VPA treatment restored GABA immunoreactivity in the somata of *Gad1* (–/–) rats (Figure 6D, left). These results are quantified and summarized in Figure 6E. GABA immunoreactivity was significantly lower in *Gad1* (–/–) rats (*P* = 7.4 × 10^−9^; Kruskal–Wallis test). The GABA immunoreactivity was restored in *Gad1* (–/–) after VPA treatment, comparable to that of *Gad1* (+/+) rats (*P* = 0.69; Wilcoxon rank-sum test).

By contrast, the patterns of GABA immunoreactivity in the RTN axons targeting the thalamus were different from those found in the RTN somata. The GABA immunoreactivity in the RTN axons was similar between *Gad1* (+/+) (Figure 6A, right) and *Gad1* (–/–) rats (Figure 6C, right). VPA treatment led to a marked increase in GABA immunoreactivity in the axons of both *Gad1* (+/+) rats (Figure 6B, right) and *Gad1* (–/–) rats (Figure 6D, right) to a similar degree. These results are summarized in Figure 6F. The difference in GABA immunoreactivity was not significantly different between the two genotypes without VPA treatment [VPA(–)] (*P* = 0.54; Wilcoxon rank-sum test) or with VPA treatment [VPA(+)] (*P* = 0.73). These results suggest that GAD1 has a less influential role in axonal GABA production. In summary, we detected a significant reduction in GABA immunoreactivity in the RTN neuronal somata in *Gad1* (–/–) rats, consistent with the notion that GAD1 is essential for GABA synthesis in the RTN. The residual GABA in *Gad1* (–/–) rats and excess GABA after VPA treatment are presumably produced by another GABA-synthesizing enzyme, GAD2, which is also expressed in RTN neurons and predominantly localized in the axons (28). The absence of functional GAD1 in the RTN and the excess GABA production in the axons after VPA treatment may account for the contradictory VPA treatment effect observed in *Gad1* (–/–) rats (see the “Discussion” section).

## 4. Discussion

In this study, we demonstrated that SWD appeared in EEGs at younger ages and more frequently in *Gad1* (–/–) rats than in control rats, indicating that GAD1 plays an important role in SWD suppression. Even control rats exhibited SWDs (Figure 2), which increased in number with age (Table 3) and were alleviated by VPA treatment (Figure 4). Both GAD1 protein and mRNA levels have been reported to decrease spontaneously in the auditory cortex and hippocampus of common laboratory rats (40, 41), suggesting that the overall efficacy of GABAergic signal transduction in the brain decreases with age and that this decrease underlies the increase in SWDs observed in older control rats.

We observed that the SWD counts per hour in *Gad1* (+/–) rats were significantly smaller than those in *Gad1* (+/+) rats (Figure 2C), contrary to the observation that the SWD phenotype in *Gad1* (+/–) rats was not significantly different from that in *Gad1* (+/+) rats (Figure 2A). One likely explanation for this unexpected order relation is that one of the *Gad1* (+/+) rats used for this experiment particularity showed a severe SWD phenotype similar to that of the *Gad1* (–/–) rat. Therefore, we do not suggest that the difference seen between *Gad1* (+/–) and *Gad1* (+/+) rats reflects a biologically meaningful difference.

SWDs have been studied in rodent models of epilepsy (4–6). Rodent models have shown that SWDs associated with behavioral immobility could be readily interrupted by external acoustic stimulations or voluntarily controlled with reward cues (9–12, 33, 42), which is consistent with our observation that most SWDs and the associated behavioral immobility were interrupted by buzzer tones (Case 1 SWD, Figure 3A). However, these findings are inconsistent with the clinical observation in human cases that loss of consciousness (absence seizure) is associated with SWDs (1–3). Previous studies have suggested that SWDs in rodents may reflect regular brain activities such as those associated with whisker twitching or attentive mu rhythm (14, 43–45). Nevertheless, we demonstrated that modest numbers of SWDs were resistant to interruption by buzzer tones (Case 2 SWD, Figure 3B) (12). Our data do not strongly suggest that these SWDs were produced by sensory adaptation or loss of motivation over time. Although our buzzer stimuli were not associated with any reward or required behavioral response, they were strong enough to suppress the ongoing SWDs in the control rats for ~95% of the SWDs, comparable to ~90% reported by Rodgers et al. (12), in which their acoustic stimuli were associated with no reward as in ours. Importantly, in our *Gad1* (–/–) rats, the same stimulus achieved suppression for ~82% of the SWDs, which was significantly lower than the control rats, supporting our interpretation that *Gad1* (–/–) rats showed more severe SWDs resistant to external sensory stimuli. Further studies are required to clarify whether Case 2 SWDs indicate true epileptic activity intermixed with Case 1 SWDs, which may represent a regular brain rhythm.

The interplay between glutamatergic excitatory inputs and GABAergic inhibitory inputs to the thalamus, RTN, and cortex plays an essential role in generating SWDs. Synchronous neuronal excitation in TC neurons is associated with the depolarization of membrane potential after prolonged hyperpolarization by GABAergic inputs from RTN neurons (19, 46–49). The excitatory activity in TC neurons is transferred to the cortex through TC projections; in turn, cortical neurons provide corticothalamic input to the subcortical RTN and thalamus as feedback. The postinhibitory rebound depolarization requires T-type Ca^2+^ channels (19, 21), which are effectively blocked by ethosuximide (ETX) (9, 31). Indeed, we observed that ETX administration fully suppressed SWDs in both *Gad1* (–/–) and *Gad1* (+/+) rats (Supplementary Figure S3), confirming the involvement of T-type Ca^2+^ channels in SWD generation in these animals. Although the responsible genetic mutations in the epilepsy model rats have not been fully identified, a mutation in the gene encoding a T-type Ca^2+^ channel subunit has been suggested (5).

**Figure 7:**
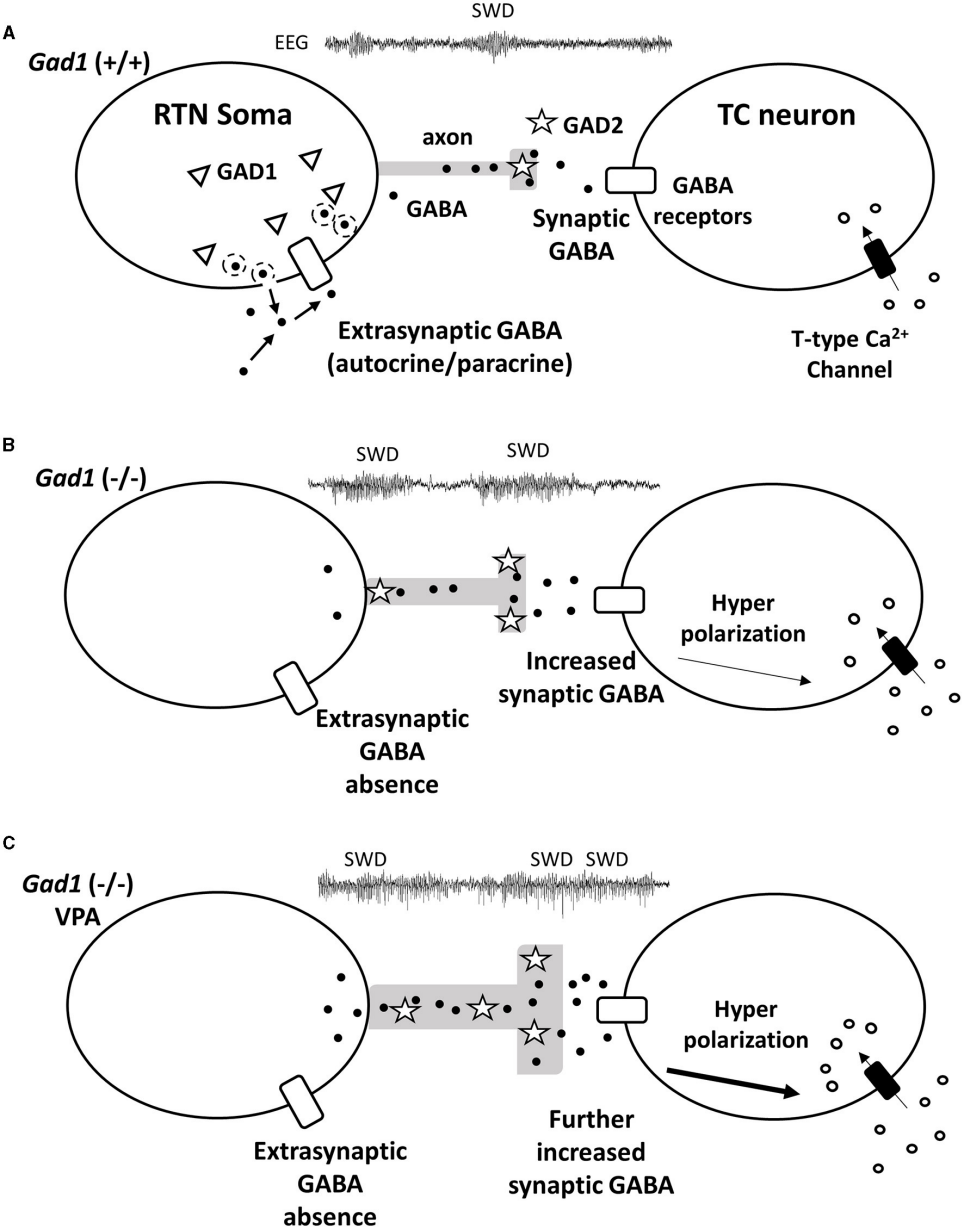
Hypothetical mechanism for GABA (black dots) signal transduction between reticular thalamic nucleus (RTN) neurons and thalamocortical (TC) relay neurons underlying SWD generation. **(A)** In the RTN neurons of *Gad1* (+/+) rats, GABA generated by GAD1 (triangles) is released extrasynaptically. The extrasynaptic GABA acts on GABA receptors (white rectangles) localized on the cell body of the RTN neuron and regulates neural activity in an autocrine/paracrine manner. **(B)** RTN neurons of *Gad1* (–/–) rats are disinhibited due to the lack of extrasynaptic GABA. Synaptic GABA synthesis is continued by intact GAD2 (stars) and is enhanced due to disinhibition. GABA binds to synaptic GABA receptors on TC neurons, resulting in deep membrane hyperpolarization and a rebound Ca^2+^ influx mediated by T-type Ca^2+^ channels. **(C)** In *Gad1* (–/–) rats with VPA treatment, synaptic GABA synthesis and release are further enhanced, resulting in profound rebound bursting of TC neurons and exacerbation of the SWD phenotype.

Our immunohistochemical experiment showed that GABA synthesis was greatly reduced in the RTN neuronal soma and minimally affected in the RTN axons in *Gad1* (–/–) rats (Figure 6). GAD2, a GAD isozyme, is also expressed in RTN neurons (28) and is likely responsible for the residual GABA synthesis. Therefore, GABAergic neurotransmission should remain functional at the inhibitory synapses on the TC neurons in *Gad1* (–/–) rats. We explored the role of GAD1 in suppressing SWD within the RTN neuronal soma. Interestingly, synaptic GABAergic connections are rare among the RTN neurons (50), and tonic inhibition mediated through extrasynaptic GABA receptors is dominant in these neurons (51). We propose that the GABA produced by GAD1 is released in an autocrine/paracrine manner from the RTN neuronal soma to inhibit RTN neurons extrasynaptically. Previous studies have demonstrated extrasynaptic GABA release in the retina (52). Our results can be accounted for by non-synaptic GABAergic inhibition within RTN neurons (Figure 7A). The GABA produced by GAD1 is released from the RTN neuronal cell bodies and maintains the basal activity of these inhibitory neurons through mutual extrasynaptic GABAergic neurotransmission. This model accounts for the enhanced SWD phenotype in *Gad1* (–/–) rats (Figure 7B). RTN neurons are tonically active (disinhibited) in these rats due to a deficiency of extrasynaptic GABA. However, GABA synthesis in the axons continues due to intact synaptic GAD2 and can even be elevated due to the compensatory increase in GAD2 expression in *Gad1* (–/–) rats (28). Therefore, GABA release at the inhibitory synapses between the disinhibited RTN axon terminals and target TC neurons should be significantly increased. The increased activation of synaptic GABA receptors on TC neurons induces deep hyperpolarization in these neurons, followed by profound rebound bursting. This rebound bursting is involved in sustained SWD generation (19, 21). A further increase in synaptic GABA production after VPA treatment (Figure 7C) results in longer and deeper hyperpolarization of TC neurons, leading to the paradoxical exacerbation of SWD symptoms in *Gad1* (–/–) rats.

In conclusion, we demonstrated that GAD1 deficiency in rats results in abundant SWDs in EEG data from an early age, comparable to other epilepsy-model rodents. *Gad1* (–/–) rats exhibited SWDs resistant to interruption by buzzer tones more frequently than control rats; therefore, we suggest that true epileptic activity may have occurred, together with regular brain rhythms. We revealed two opposing roles for GABA: suppression and enhancement of SWD generation. Our interpretation of these results depends on the hypothetical extrasynaptic GABA produced by GAD1 from the RTN neuronal soma. Further molecular and neurophysiological experiments are necessary to confirm the role played by GAD1 in extrasynaptic GABAergic transduction in the RTN.

## Data availability statement

The original contributions presented in the study are included in the article/Supplementary material, further inquiries can be directed to the corresponding author.

## Ethics statement

The animal study was approved by Tohoku University Committee for Animal Research. The study was conducted in accordance with the local legislation and institutional requirements.

## Author contributions

DL and TO designed the research, analyzed the data, and wrote the manuscript. DL conducted the experiments and collected the data. All authors contributed to the article and approved the submitted version.

## References

[1] Blumenfeld H. Consciousness and epilepsy: why are patients with absence seizures absent? Prog Brain Res. (2005) 150:271–86. 10.1016/S0079-6123(05)50020-7 16186030 PMC3153469

[2] Blumenfeld H. Epilepsy and the consciousness system: transient vegetative state? Neurol Clin. (2011) 29:801–23. 10.1016/j.ncl.2011.07.014 22032662 PMC3432285

[3] Remi J Vollmar C Noachtar S. No response to acoustic stimuli: absence or akinetic seizure? Epilep Behav. (2011) 21:478–9. 10.1016/j.yebeh.2011.06.008 21733759

[4] Gilles van Luijtelaar TC. “Genetic models of absence epilepsy in the rat,” In: Models of Seizures and Epilepsy. (2006) p. 233-48.34782347

[5] Coenen AM Van Luijtelaar EL. Genetic animal models for absence epilepsy: a review of the WAG/Rij strain of rats. Behav Genet. (2003) 33:635–55. 10.1023/a:1026179013847 14574120

[6] Danober L Deransart C Depaulis A Vergnes M Marescaux C. Pathophysiological mechanisms of genetic absence epilepsy in the rat. Prog Neurobiol. (1998) 55:27–57. 10.1016/S0301-0082(97)00091-9 9602499

[7] Shaw FZ. 7-12 Hz high-voltage rhythmic spike discharges in rats evaluated by antiepileptic drugs and flicker stimulation. J Neurophysiol. (2007) 97:238–47. 10.1152/jn.00340.2006 17035363

[8] Taylor JA Reuter JD Kubiak RA Mufford TT Booth CJ Dudek FE. Spontaneous recurrent absence seizure-like events in wild-caught rats. J Neurosci. (2019) 39:4829–41. 10.1523/JNEUROSCI.1167-18.2019 30971439 PMC6561690

[9] Taylor JA Rodgers KM Bercum FM Booth CJ Dudek FE Barth DS. Voluntary control of epileptiform spike-wave discharges in awake rats. J Neurosci. (2017) 37:5861–9. 10.1523/JNEUROSCI.3235-16.2017 28522734 PMC6596506

[10] Saillet S Gharbi S Charvet G Deransart C Guillemaud R Depaulis A. Neural adaptation to responsive stimulation: a comparison of auditory and deep brain stimulation in a rat model of absence epilepsy. Brain Stimul. (2013) 6:241–7. 10.1016/j.brs.2012.05.009 22727526

[11] Drinkenburg WHIM Schuurmans MLEJ Coenen AML Vossen JMH van Luijtelaar ELJM. Ictal stimulus processing during spike-wave discharges in genetic epileptic rats. Behav Brain Res. (2003) 143:141–6. 10.1016/S0166-4328(03)00031-7 12900040

[12] Rodgers KM Dudek FE Barth DS. Progressive, seizure-like, spike-wave discharges are common in both injured and uninjured Sprague-Dawley rats: implications for the fluid percussion injury model of post-traumatic epilepsy. J Neurosci. (2015) 35:9194–204. 10.1523/JNEUROSCI.0919-15.2015 26085641 PMC6605152

[13] Shaw FZ. Is spontaneous high-voltage rhythmic spike discharge in Long Evans rats an absence-like seizure activity? J Neurophysiol. (2004) 91:63–77. 10.1152/jn.00487.2003 12826656

[14] Wiest MC Nicolelis MA. Behavioral detection of tactile stimuli during 7-12 Hz cortical oscillations in awake rats. Nat Neurosci. (2003) 6:913–4. 10.1038/nn1107 12897789

[15] Gorji A Mittag C Shahabi P Seidenbecher T Pape HC. Seizure-related activity of intralaminar thalamic neurons in a genetic model of absence epilepsy. Neurobiol Dis. (2011) 43:266–74. 10.1016/j.nbd.2011.03.019 21458572

[16] Hu B Guo Y Shi F Zou X Dong J Pan L. The generation mechanism of spike-and-slow wave discharges appearing on thalamic relay nuclei. Sci Rep. (2018) 8:4953. 10.1038/s41598-018-23280-y 29563579 PMC5862852

[17] Meeren HK Pijn JP Van Luijtelaar EL Coenen AM Lopes da Silva FH. Cortical focus drives widespread corticothalamic networks during spontaneous absence seizures in rats. J Neurosci. (2002) 22:1480–95. 10.1523/JNEUROSCI.22-04-01480.2002 11850474 PMC6757554

[18] Sitnikova E. Thalamo-cortical mechanisms of sleep spindles and spike-wave discharges in rat model of absence epilepsy (a review). Epilepsy Res. (2010) 89:17–26. 10.1016/j.eplepsyres.2009.09.005 19828296

[19] Fogerson PM Huguenard JR. Tapping the brakes: cellular and synaptic mechanisms that regulate thalamic oscillations. Neuron. (2016) 92:687–704. 10.1016/j.neuron.2016.10.024 27883901 PMC5131525

[20] Pinault D. The thalamic reticular nucleus: structure, function and concept. Brain Res Rev. (2004) 46:1–31. 10.1016/j.brainresrev.2004.04.008 15297152

[21] Leresche N Lambert RC. GABA receptors and T-type Ca^2+^ channels crosstalk in thalamic networks. Neuropharmacology. (2018) 136:37–45. 10.1016/j.neuropharm.2017.06.006 28601398

[22] Wu H Jin Y Buddhala C Osterhaus G Cohen E Jin H. Role of glutamate decarboxylase (GAD) isoform, GAD65, in GABA synthesis and transport into synaptic vesicles-evidence from GAD65-knockout mice studies. Brain Res. (2007) 1154:80–3. 10.1016/j.brainres.2007.04.008 17482148

[23] Walls AB Eyjolfsson EM Smeland OB Nilsen LH Schousboe I Schousboe A. Knockout of GAD65 has major impact on synaptic GABA synthesized from astrocyte-derived glutamine. J Cereb Blood Flow Metab. (2011) 31:494–503. 10.1038/jcbfm.2010.115 20664610 PMC3049505

[24] Stork O Ji FY Kaneko K Stork S Yoshinobu Y Moriya T. Postnatal development of a GABA deficit and disturbance of neural functions in mice lacking GAD65. Brain Res. (2000) 865:45–58. 10.1016/S0006-8993(00)02206-X 10814732

[25] Kakizaki T Ohshiro T Itakura M Konno K Watanabe M Mushiake H. Rats deficient in the GAD65 isoform exhibit epilepsy and premature lethality. FASEB J. (2021) 35:e21224. 10.1096/fj.202001935R 33236473

[26] Qi J Kim M Sanchez R Ziaee SM Kohtz JD Koh S. Enhanced susceptibility to stress and seizures in GAD65 deficient mice. PLoS ONE. (2018) 13:e0191794. 10.1371/journal.pone.0191794 29377906 PMC5788371

[27] Esclapez M TN Kaufman DL Tobin AJ Houser CR. Comparative. Comparative localization of two forms of glutamic acid decarboxylase and their mRNAs in rat brain supports the concept of functional differences between the forms. Neuroscience. (1994) 14:834–1855. 10.1523/JNEUROSCI.14-03-01834.1994 8126575 PMC6577546

[28] Panthi S Lyons NMA Leitch B. Impact of dysfunctional feed-forward inhibition on glutamate decarboxylase isoforms and gamma-aminobutyric acid transporters. Int J Mol Sci. (2021) 22:14. 10.3390/ijms22147740 34299369 PMC8306481

[29] Fujihara K Yamada K Ichitani Y Kakizaki T Jiang W Miyata S. CRISPR/Cas9-engineered *Gad1* elimination in rats leads to complex behavioral changes: implications for schizophrenia. Transl Psychiatry. (2020) 10:426. 10.1038/s41398-020-01108-6 33293518 PMC7723991

[30] Kuki T Ohshiro T Ito S Ji ZG Fukazawa Y Matsuzaka Y. Frequency-dependent entrainment of neocortical slow oscillation to repeated optogenetic stimulation in the anesthetized rat. Neurosci Res. (2013) 75:35–45. 10.1016/j.neures.2012.10.007 23154073

[31] Loscher W. The pharmacokinetics of antiepileptic drugs in rats: consequences for maintaining effective drug levels during prolonged drug administration in rat models of epilepsy. Epilepsia. (2007) 48:1245–58. 10.1111/j.1528-1167.2007.01093.x 17441999

[32] Fang G Xia Y Zhang C Liu T Yao D. Optimized single electroencephalogram channel sleep staging in rats. Lab Anim. (2010) 44:312–22. 10.1258/la.2010.009081 20610470

[33] Vergnes M Marescaux C Boehrer A Depaulis A. Are rats with genetic absence epilepsy behaviorally impaired? Epilepsy Res. (1991) 9:97–104. 10.1016/0920-1211(91)90019-C 1794357

[34] Watanabe Y Takechi K Fujiwara A Kamei C. Effects of antiepileptics on behavioral and electroencephalographic seizure induced by pentetrazol in mice. J Pharmacol Sci. (2010) 112:282–9. 10.1254/jphs.09225FP 20168048

[35] Loscher W. Basic pharmacology of valproate—a review after 35 years of clinical use for the treatment of epilepsy. CNS Drugs. (2002) 16:669–94. 10.2165/00023210-200216100-00003 12269861

[36] Kawaguchi Y Kondo S. Parvalbumin, somatostatin and cholecystokinin as chemical markers for specific GABAergic interneuron types in the rat frontal cortex. J Neurocytol. (2002) 31:277–87. 10.1023/A:1024126110356 12815247

[37] Kajita Y Mushiake H. Heterogeneous GAD65 Expression in subtypes of GABAergic neurons across layers of the cerebral cortex and hippocampus. Front Behav Neurosci. (2021) 15:750869. 10.3389/fnbeh.2021.750869 34803625 PMC8595203

[38] Steullet P Cabungcal JH Bukhari SA Ardelt MI Pantazopoulos H Hamati F. The thalamic reticular nucleus in schizophrenia and bipolar disorder: role of parvalbumin-expressing neuron networks and oxidative stress. Mol Psychiatry. (2018) 23:2057–65. 10.1038/mp.2017.230 29180672 PMC5972042

[39] Williams SM Goldman-Rakic PS Leranth C. The synaptology of parvalbumin-immunoreactive neurons in the primate prefrontal cortex. J Comp Neurol. (1992) 320:353–69. 10.1002/cne.903200307 1613130

[40] Ling LL Hughes LF Caspary DM. Age-related loss of the GABA synthetic enzyme glutamic acid decarboxylase in rat primary auditory cortex. Neuroscience. (2005) 132:1103–13. 10.1016/j.neuroscience.2004.12.043 15857714

[41] Stanley DP Shetty AK. Aging in the rat hippocampus is associated with widespread reductions in the number of glutamate decarboxylase-67 positive interneurons but not interneuron degeneration. J Neurochem. (2004) 89:204–16. 10.1111/j.1471-4159.2004.02318.x 15030405

[42] Li X Ouyang G Richards DA. Predictability analysis of absence seizures with permutation entropy. Epilepsy Res. (2007) 77:70–4. 10.1016/j.eplepsyres.2007.08.002 17870413

[43] Shaw FZ Liao YF. Relation between activities of the cortex and vibrissae muscles during high-voltage rhythmic spike discharges in rats. J Neurophysiol. (2005) 93:2435–48. 10.1152/jn.00999.2004 15625092

[44] Nicolelis MA Fanselow EE. Thalamocortical correction of thalamcortical optimization of tactile processing according to behavioral state. Nat Neurosci. (2002) 5:517–23. 10.1038/nn0602-517 12037519

[45] Shaw FZ Liao YF Chen RF Huang YH Lin RC. The zona incerta modulates spontaneous spike-wave discharges in the rat. J Neurophysiol. (2013) 109:2505–16. 10.1152/jn.00750.2011 23446687

[46] Guillery RW Feig SL Lozsadi DA. Paying attention to the thalamic reticular nucleus. Trends Neurosci. (1998) 21:28–32. 10.1016/S0166-2236(97)01157-0 9464683

[47] Clemente-Perez A Makinson SR Higashikubo B Brovarney S Cho FS Urry A. Distinct thalamic reticular cell types differentially modulate normal and pathological cortical rhythms. Cell Rep. (2017) 19:2130–42. 10.1016/j.celrep.2017.05.044 28591583 PMC5557038

[48] Schofield CM Kleiman-Weiner M Rudolph U Huguenard JR A. gain in GABAA receptor synaptic strength in thalamus reduces oscillatory activity and absence seizures. Proc Natl Acad Sci U S A. (2009) 106:7630–5. 10.1073/pnas.0811326106 19380748 PMC2678654

[49] Huntsman MM Porcello DM Homanics GE DeLorey TM Huguenard JR. Reciprocal inhibitory connections and network synchrony in the mammalian thalamus. Science. (1999) 283:541–3. 10.1126/science.283.5401.541 9915702

[50] Hou G Smith AG Zhang ZW. Lack of intrinsic GABAergic connections in the thalamic reticular nucleus of the mouse. J Neurosci. (2016) 36:7246–52. 10.1523/JNEUROSCI.0607-16.2016 27383598 PMC4938865

[51] Crabtree JW Lodge D Bashir ZI Isaac JT GABAA. NMDA and mGlu2 receptors tonically regulate inhibition and excitation in the thalamic reticular nucleus. Eur J Neurosci. (2013) 37:850–9. 10.1111/ejn.12098 23294136 PMC4243027

[52] Hirasawa H Contini M Raviola E. Extrasynaptic release of GABA and dopamine by retinal dopaminergic neurons. Philos Trans R Soc Lond B Biol Sci. (2015) 370:1672. 10.1098/rstb.2014.0186 26009765 PMC4455755

