# Supplementary material for: Gad1 knock-out rats exhibit abundant spike-wave discharges in EEG, exacerbated with valproate treatment
Source: Front Neurol. 2023 Sep 26;14:1243301. doi: 10.3389/fneur.2023.1243301 (PMC10566305; doi:10.3389/fneur.2023.1243301)
Supplement: Supplementary file 1 [file Presentation_1.pdf]

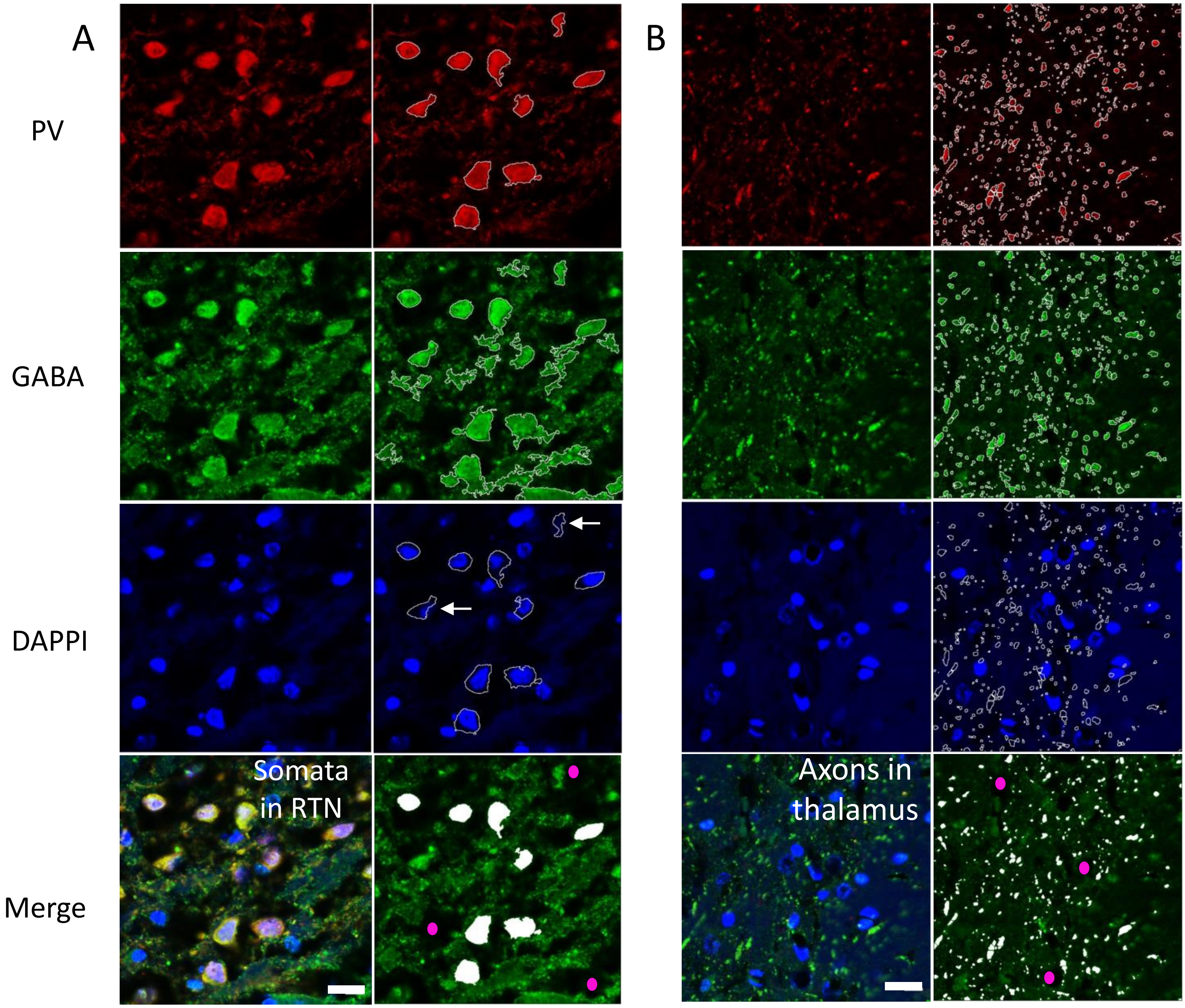

Figure S1 Detection and quantification of gamma-aminobutyric acid (GABA) localized in reticular thalamic nucleus (RTN) neuronal soma and axons targeting the thalamus in a wild-type *Gad1* homozygous (+/+) rat. ImageJ software was used to define the region of interest (ROI) for the cell soma and axons. **(A)** In the RTN, neuron soma-like signals were first detected (white circles) in the parvalbumin (PV, red) and GABA (green) signal channels; only overlapped areas were automatically selected. False-positive contours without a clear nucleus indicated by 4',6-diamidino-2-phenylindol (DAPI, blue) were removed by visual inspection (arrows). Neuronal somata (white areas) and background (magenta dots) intensities were obtained from the green GABA signal channel. The GABA signal intensities without background interference were obtained by subtracting the mean intensity of the background from each image. Approximately five somata were detected in each RTN image. **(B)** In the thalamus, axon-like signals were detected in both channels. Overlapping contours were automatically retained as ROIs. ROIs were manually removed if the contour included a DAPI signal. Approximately 800 ROIs were obtained in each image. Average signal intensity was compared among groups (main figures 5D, E). White scale bar: 20 µm.

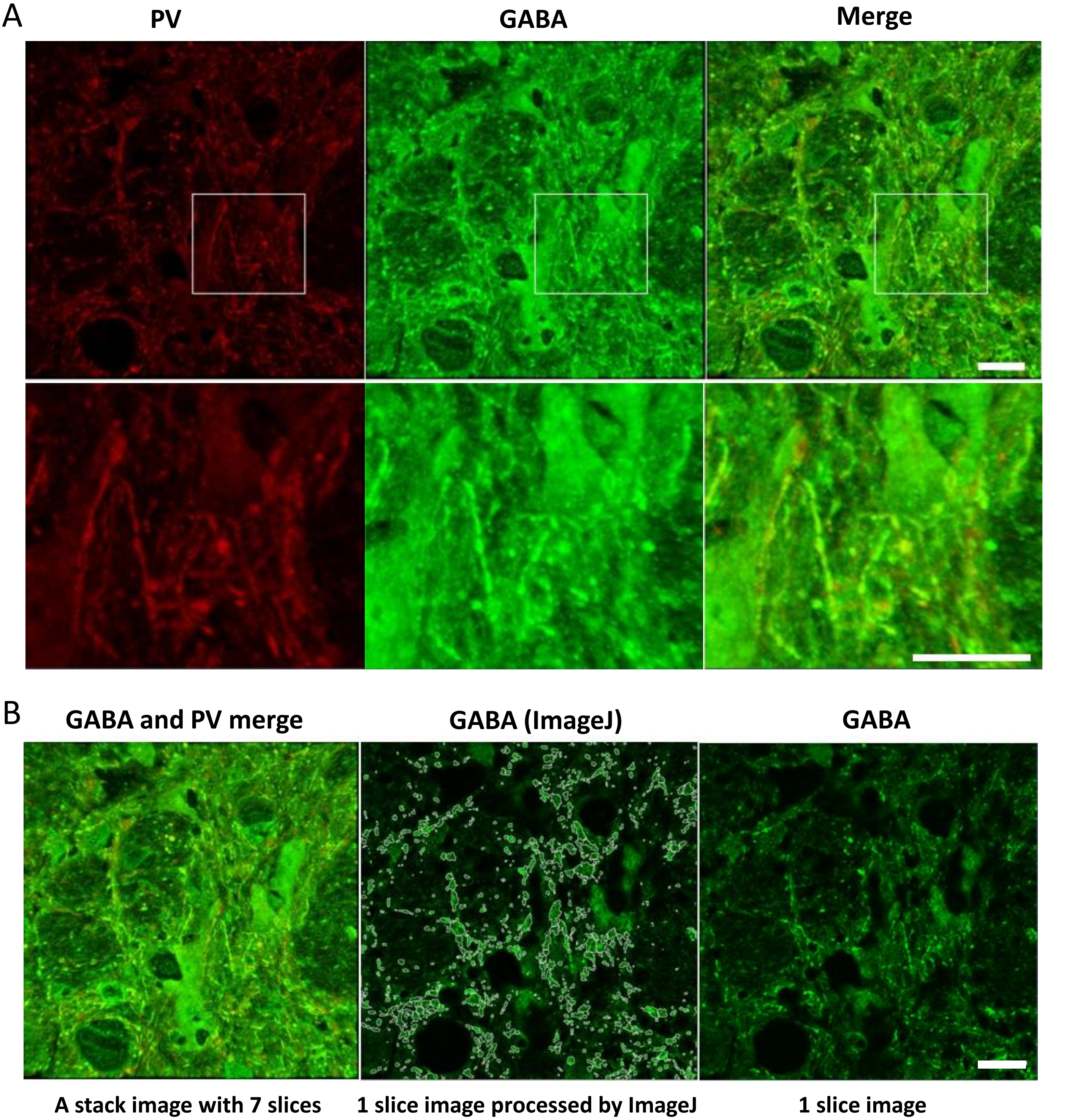

Figure S2 Punctate GABA signals in axons were distinguished from the background. **(A)** Punctate signals corresponding to synaptic vesicles containing neurotransmitters, GABA, and parvalbumin (PV) transported along the RTN axons were clearly identified on the stacked, optically sliced images. The framed section in each channel is enlarged at bottom. **(B)** Compared with the stacked thalamus image (left), a single optical slice image often gives rise to relatively isolated, fragmented GABA signals (right). These bright punctate signals were still clearly distinguished from the vague background signal by the ImageJ software (middle). White scale bar: 20 μm.

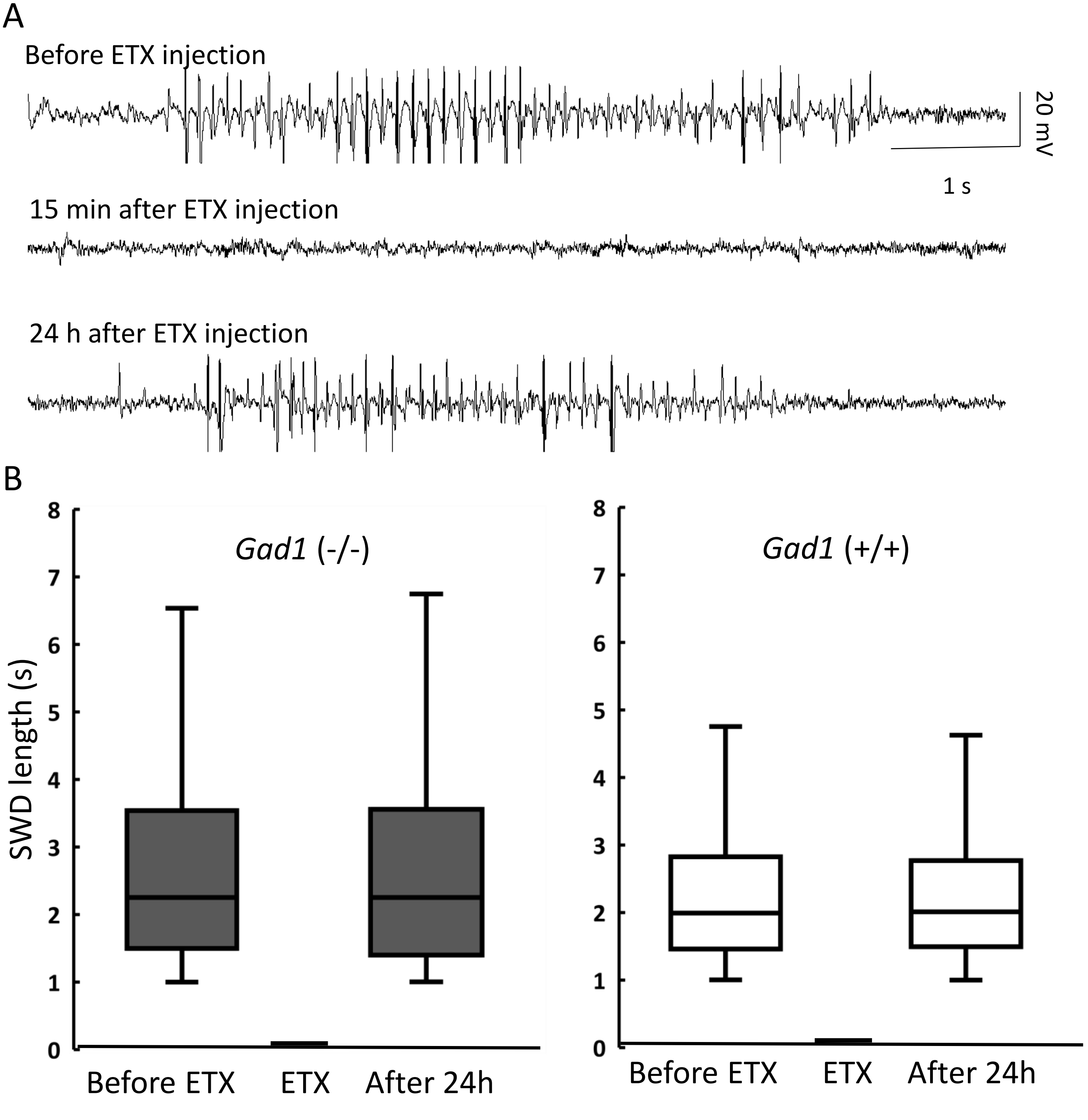

Figure S3 Comparison of spike-wave discharges (SWDs) before, during, and after intraperitoneal injection of ethosuximide (ETX). (A) Electroencephalogram (EEG) patterns from a *Gad1* homozygous (–/–) knockout rat. The *Gad1* (+/+) rats showed a similar pattern. Pre-medication EEG recording was performed 3–4 h prior to intraperitoneal ETX injection (250 mg/kg). Recording was resumed 15 min after the injection. ETX completely suppressed SWDs for 6–8 h. SWDs returned after 24 h. (B) Box plots of SWD length. There were no significant differences between before and after ETX administration in either *Gad1* (–/–) rats (n = 346 SWDs before ETX; n = 491 SWDs after 24 h; *P* = 0.73; Wilcoxon's rank-sum test) or *Gad1* (+/+) rats (n = 290 SWDs before ETX; 403 SWDs after 24 h; *P* = 0.96; Wilcoxon's rank-sum test). In total, 16 h of EEG data were obtained and analyzed from *Gad1* (–/–) rats (n = 2, age: 3 and 6 months), and 12 h from two *Gad1* (+/+) rats (n = 2, age: 5 and 7 months).
